# Multipath Propagation of Acoustic Signal in a Swimming Pool—Source Localization Problem †

**DOI:** 10.3390/s22031162

**Published:** 2022-02-03

**Authors:** Jacek Misiurewicz, Konrad Bruliński, Wiesław Klembowski, Krzysztof Stefan Kulpa, Jan Pietrusiewicz

**Affiliations:** 1 Institute of Electronic Systems, Warsaw University of Technology, 00-665 Warsaw, Poland; krzysztof.kulpa@pw.edu.pl (K.S.K.); jan.pietrusiewicz.stud@pw.edu.pl (J.P.); 2 Berserg sp. z o.o., 87-100 Toruń, Poland; 123konrad@gmail.com (K.B.); klembowskiw@gmail.com (W.K.)

**Keywords:** multipath propagation, underwater propagation modeling, detection, TDOA, multi-hypothesis testing

## Abstract

This paper explores the problem of severe multipath propagation of underwater acoustic signals in a swimming pool. The problem appeared in a study that examined a system used to signal emergency situations (i.e., pre-drowning symptoms detected by a wearable device on a pool user’s wrist) and locate the signal source. A swimming pool acoustic environment is characterized by the presence of large flat reflecting planes surrounding a small volume of water. The reflections are numerous and much stronger than in typical hydroacoustic applications. In this paper, we attempted to create a model of the swimming pool response, one that is suitable for simulation experiments with detection and localization of emergency signals. Then, we explore the possible remedies for the localization system, applied on the transmit side (waveform design) and on the receive side (receiver placement and signal processing). Finally, we present an algorithm for object localization, considering the possible reflections with a multi-hypothesis approach.

## 1. Introduction

The multipath propagation problem appeared in connection with a study of an underwater signaling system designed for the safety of public swimming pools. The system’s concept required reliable detection of a distress signal as well as accurate measurements of the signal’s arrival time. The latter feature was intended to be used to locate the signal source with the time difference of arrival (TDOA) technique.

According to WHO statistics, drowning is the third leading cause of unintentional injury death worldwide, accounting for 7% of all injury-related deaths [1]. However the majority of drowning incidents happen in unsupervised waters; incidents in private and public swimming pools are most easily prevented. This has led to a strong interest in "in-water monitoring of users" methods in these objects. One known method is the usage of special wristbands, which monitor the depth and time of staying underwater. Such a wristband generates an alarm signal upon detecting an emergency (i.e., potential user drowning). A logical choice for signaling in water is the use of hydroacoustic signaling [2]. The signal, received by hydrophones installed in the pool walls, is transmitted to the pool staff room (the rescue team) and it triggers the rescue action.

A rescue delay, in a situation where an individual is drowning, may mean the difference between life and death. Thus, good localization of the alarm source is required, especially in large pool installations.

In the presence of multipath propagation phenomena that may distort volume characteristics of the receiving hydrophones, the amplitude-phase methods of direction estimations will be affected by high measurement errors, which seriously limit their applications. A more helpful method for locating the alarm source is TDOA, as it is based on the estimation of the reception time difference in the hydrophones.

Much research efforts have been devoted toward the acoustic environment of a sea or an ocean. Some of the works worth noting have dealt with limitations on sonar performance due to multipath effects [3]. One technique of multipath component estimation is presented in [4]. Moreover, emitter localization [5] and tracking [6] have been widely studied.

It should be noted that the sea environment [7] is mainly characterized by refraction effects due to different properties of water layers, changing with temperature and pressure. The reflection component is present, but it is not a dominating one. A swimming pool acoustic environment differs from the sea environment by the presence of large, flat reflecting planes, and a small volume of water, usually in cuboid-like shapes. As a result, the reflections are numerous and very strong. In regard to pools that are heavily used, there are significant levels of noise generated by pumps, water filters, and amusement attractions, such as cascades and geysers, as well as noise produced by users. As detecting a signal in such an environment should be performed reliably, and with time-of-arrival estimation, one must deal with overlaying echoes. The problem is especially severe when the transmit power is limited, and the pulse compression must be used with a long pulse. Then, many pulse copies overlay within the compression side lobe area, making constant false alarm rate (CFAR) processing difficult.

Another problematic situation that we observed, in practice, occurred when the straight path to a given receiver exhibited high attenuation. In such a setup, the first detectable impulse may come from one of the bounce paths for a particular receiver and via the straight path for other receivers. This makes the time-of-arrival measurement set inconsistent with the actual location of the source. We observed such problems when the source was very close to the same wall where the receiver was mounted; thus, we suspect that the propagation along a wall is impaired, for some reasons; however, the study of this phenomenon is outside the scope of this paper.

The TDOA technique for source localization is based on measuring signal arrival times at separate receivers. When the signals arrive via different paths—directly or with a reflection—the measurements at one or more sensors may correspond to the reflection. If it is mistaken for the direct path measurement, the location result is wrong. Several solutions to this problem were proposed in the literature (see the review in Section 5.3), but they are not suitable for a swimming pool environment.

In the first section of the paper, we present a geometrical model of the swimming pool. Then, an impulse response is modeled and verified with live hydroacoustic recordings from real pools. A simple model assumes idealistic cuboidal geometry, which is usually inaccurate due to several factors, including the sloped pool bottom in a typical installation. Thus, a random model was constructed to avoid a complex geometrical analysis.

Then, we studied a waveform design to mitigate the problems and propose improvements in detection methodology.

The second section of the paper is devoted to a multi-hypothesis TDOA algorithm, which attempts to accommodate the possibility of receiving the reflected signal instead of the direct one.

This article is a post-conference publication. The original conference paper [8] deals with modeling the pool response and the reception of the signal, whereas the present article adds to the description of the TDOA methodology modification.

## 2. Measured Examples

**Figure 1:**
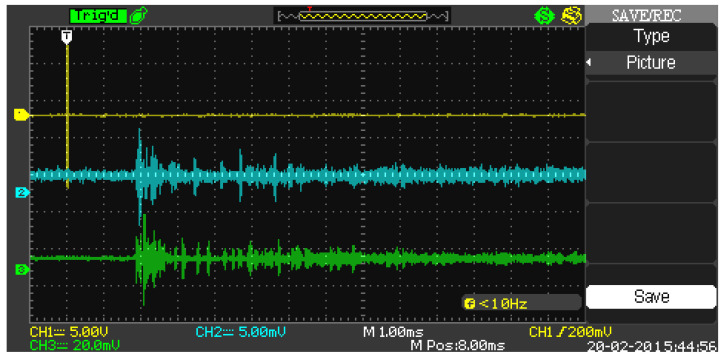
Example of measured signal [9]; trace 1 (yellow)—transmitted signal, trace 2 (blue), and 3 (green)—recorded signals from different receiver locations, showing different distributions of signal peaks due to the multitude of propagation paths. The difference in signal magnitudes is the result of using different receiver setups in both channels.

**Figure 2:**
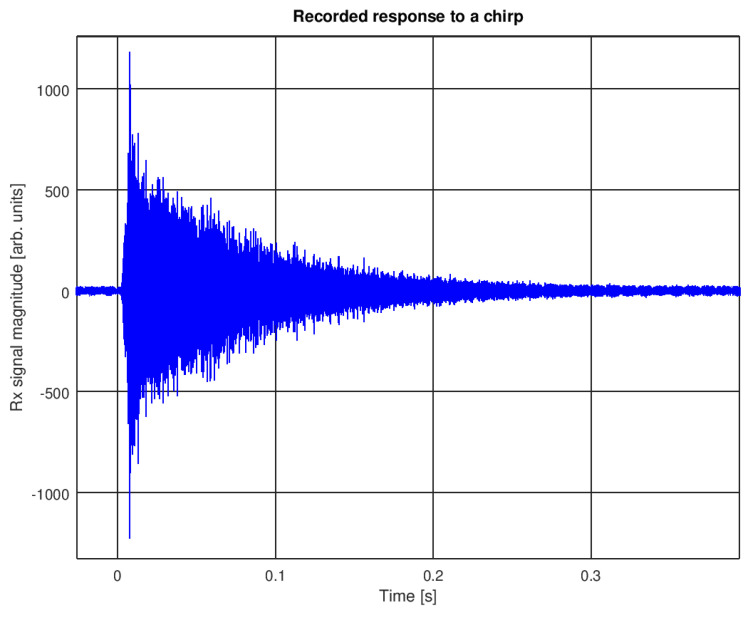
Decay example with chirp signal (garden-type pool).

An interesting measurement set was presented in [9], where a short modulated pulse was transmitted, and the signal with a significant echo was recorded in a small (garden-type, 8 × 10 m in size and approximately 1 m deep) swimming pool. A different example with the same pool was presented in (Figure 1), with a short time scale: a three-period pulse at 80 kHz (trace 1) was received by two hydrophones located approximately 3 m from the transmitter in different directions. The transmitter and receivers were 25–35 cm under the water surface. The transmitted and received signals were visualized on an oscilloscope screen. Another example with a chirp signal (4 ms long, 50–80 kHz LFM) and with a longer time scale shows that the echo decays to the noise level after approximately 0.25 s (Figure 2). The exponential decay time constant estimated from several such measurements is approximately equal to 0.125 s. One can also notice a delay of 2.5 ms corresponding to the transmitter–receiver distance, of 3.75 m, followed by 4 ms of magnitude increase, due to “pumping” the energy into the pool during the transmit active time. The recordings were made with a dedicated pool—no swimmers were present, all of the pool equipment was switched off.

## 3. Modeling the Swimming Pool Environment

In an effort to model the multipath environment of a swimming pool (presented below), the first step involves defining the pool geometry. For the sake of simplicity, the drawings present a two-dimensional model, but the mathematical derivation and MATLAB modeling results refer to a three-dimensional setup.

In the following, we derive a simple mathematical description for a case when the pool is represented by a rectangular cuboid. Then, we proceed to more complex pool shapes, by a random shape model. It will be shown that, despite the model simplicity, the signal modeled with the latter model resembles the measurement results pretty well.

### 3.1. Geometrical Model

**Figure 3:**
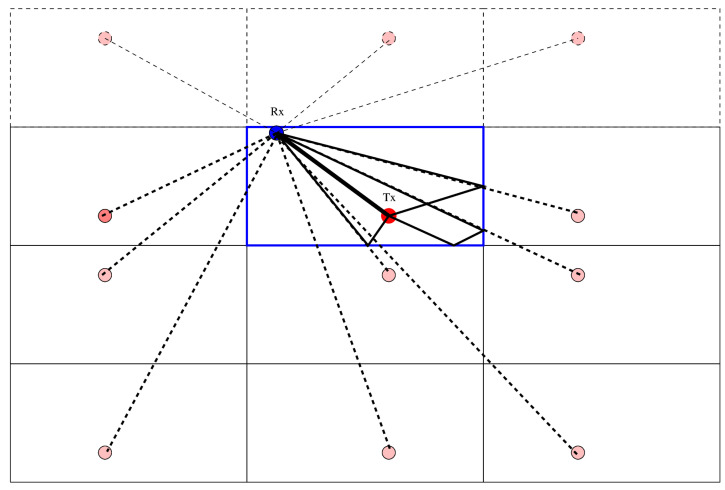
Schematic of multipath reflection (2D).

Let us imagine a signal source (Tx mark in the 2D drawing at Figure 3), located in an arbitrary position within a rectangular swimming pool (blue rectangle), and a signal receiver (Rx mark). The shortest path from Tx to Rx is the straight path. Large pool wall planes reflect the signal with the geometrical reflection (mirror) rule; the pool bottom and water surface in a 3D case also act as reflecting surfaces. Due to the large ratio of the wave velocity in the water and air (approximately 4.3×) or water and concrete (approximately 2.2×), a significant part of wave energy is reflected. In the former case (water/air), the reflection coefficient is negative (i.e., sign change occurs); in the latter—it is positive. A specific case involves a rubberized fabric wall of a garden pool, where the fabric is thin—this case can be effectively considered a water/air reflection.

The length of the reflection path is equal to a path from a mirrored source, where the “mirror” is the plane that reflects the signal. The drawing shows all of the mirrored sources for one bounce and some sources for two bounces with pink spots. Please note that the solid black true paths are not shown for all cases.

The drawing in Figure 3 shows the 2D case; for the practical pool with bottom and surface reflections, it is actually a 3D case. We consider it easy to imagine but difficult to present in a legible drawing.

### 3.2. Reflections—Deterministic Model

This graphical representation allows us to present a simple model for a rectangular cuboid pool impulse response. This model takes a form

(1) $$h\left(t\right)=\sum_{n}^{N}A_{n}\delta \left(t-\tau_{n}\right)$$

where $\left(A_{n},\tau_{n}\right)$ pairs correspond to the magnitude and delay of a signal coming from the *n*-th path, and they are found from an obvious (but tedious) formula for the Tx_*n*_—Rx distance, iterating through all the *N* considered positions of the mirror sources (including the real source).

The number *N* of considered sources depends on the accuracy required and on the assumed attenuation of the signal. For our simulation experiments, we matched this to the observations from a test pool showing that an impulse signal decays approximately in 0.25 s, which corresponds to a path of l= 375 m in water.

Cutting off at *l*, we should consider all of the mirrored sources within the distance of *l* from the receiver. Thus, for a test pool size 8×4×1.5 m, there will be $N\approx \frac{4/3\pi l^{3}}{V_{pool}}$≈$\frac{4\cdot 375^{3}}{8\cdot 4\cdot 1.5}$≈ 4,000,000 sources in the model.

In general, one may expect fixed attenuation at each reflection and some attenuation in the propagation path. Our modeling experiments assumed an overall attenuation of 30 dB/km (0.03 dB/m), including both reflection and propagation contribution to the signal damping—“all in one”.

Together, our model includes calculations of each *n*-th distance:

(2) $$r_{n}=\left|x_{Rx}-x_{Tx_{n}}\right|$$

where $x_{Rx}$ and $x_{Tx_{n}}$ denote a 3D location vector of the receiver or transmitter in Cartesian coordinates, finding the coefficients

(3) $$A_{n}=\pm 10^{-r_{n}\cdot 0.03/20}\cdot \frac{1}{4\pi r_{n}^{2}}.$$

The $A_{n}$ coefficient includes both the “overall” attenuation and free-space propagation factor. Please note that each reflection from the water/air interface changes the sign of the reflected wave, so the sign of the $A_{n}$ coefficient should be set accordingly (i.e., negative if the number of water/air reflections is odd). Finally, we translate the distance scale to time scale:

(4) $$\tau_{n}=r_{n}\cdot v$$

where the speed of sound in water *v* is assumed 1500 m/s.

The primary deficiency of the presented model is the assumption of perfectly parallel walls and perfectly geometric reflections. In practice, neither is 100% true, and the causes may be uneven surfaces, depth differences along the pool, construction errors, water streams, and many others. As our system uses wavelengths in the centimeter range, even slight variations in the geometry may influence the impulse response, especially in the multi-bounce area (i.e., large delay).

Additionally, our laboratory test pool is not of a cuboid shape at all. All of these factors encouraged us to propose another model, representing the reflection statistically.

### 3.3. Reflections—Random Model

The random model of the pool impulse response is based on similar principles as the Rayleigh fading model, which is well-known in the telecommunications community. The calculated lengths of signal paths from the deterministic model are now replaced with $r_{n}$ values drawn randomly, but the attenuation coefficients $A_{n}$ still follow the Equation (3). Such a model is not bound to a single, deterministic location of the transmitter and receiver. Thus, it may be used in the analytical form to represent all possible configurations or simulations to draw conclusions from the result statistics.

The distribution of random $r_{n}$ values should follow the analysis of pool geometry, adapted from Section 3.1. As the number N(r) of paths with lengths up to *r* follows approximately the ratio of volumes of a ball with radius *r* and of the pool cuboid

(5) $$N\left(r\right)\approx \frac{4/3\pi r^{3}}{V_{pool}},$$

the cumulative distribution of the $r_{n}$ path lengths should also follow this relation.

Thus, a density of $r_{n}$ should be a quadratic function of *r*. It must be noted that such a function is not bounded, so it must be considered together with the attenuation and propagation effects (3), which together set a limit on the practical range of *r* considered.

Finally, the $A_{n}$ coefficients should be assigned to each of the paths according to (3). The sign of each $A_{n}$ may also be chosen in a probabilistic manner—with sufficiently large *n*, the chances for an even or odd number of water/air reflections are equal.

The approximation (5) is realistic for *r* larger than the pool size; in the small *r* range, the model should be modified by removing components shorter than the expected direct path. Moreover, one can consider using the deterministic model for the lower *n* values.

**Figure 4:**
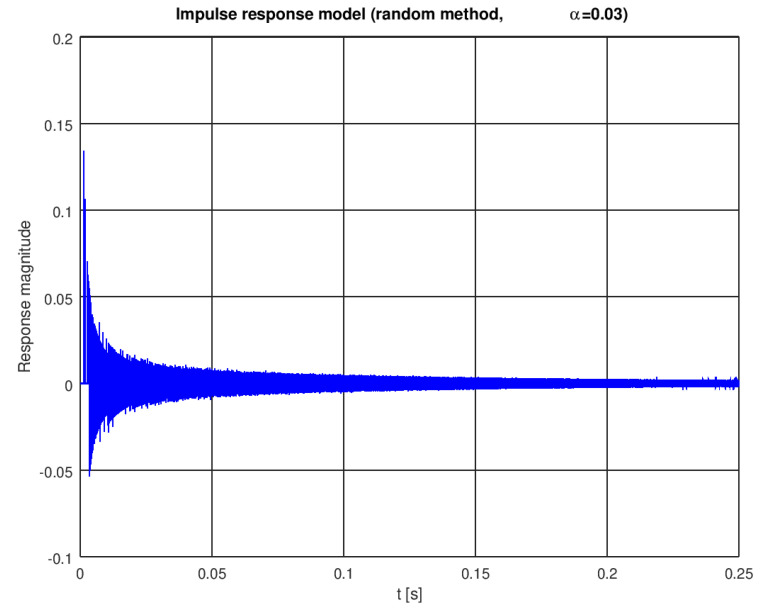
Impulse response of a pool—effect of the random modeling.

**Figure 5:**
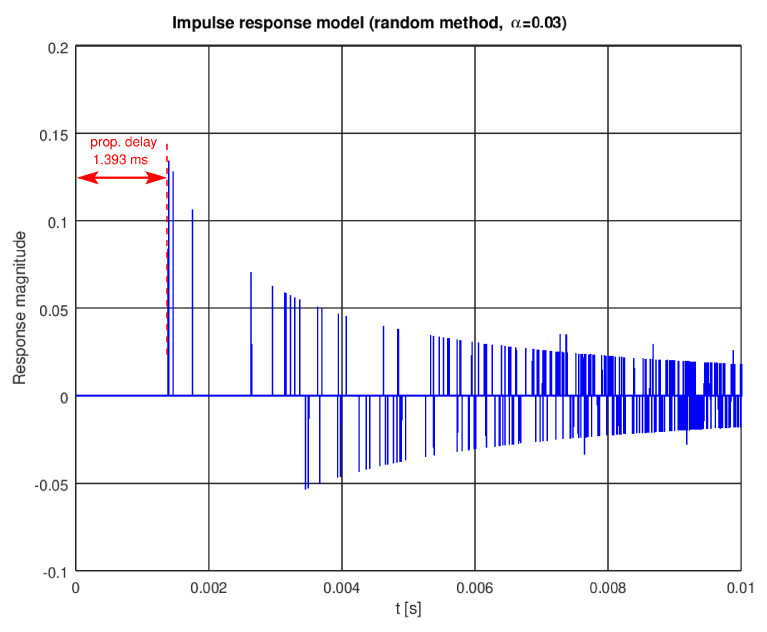
Zoomed initial part of Figure 4 (impulse response with the random modeling).

**Figure 6:**
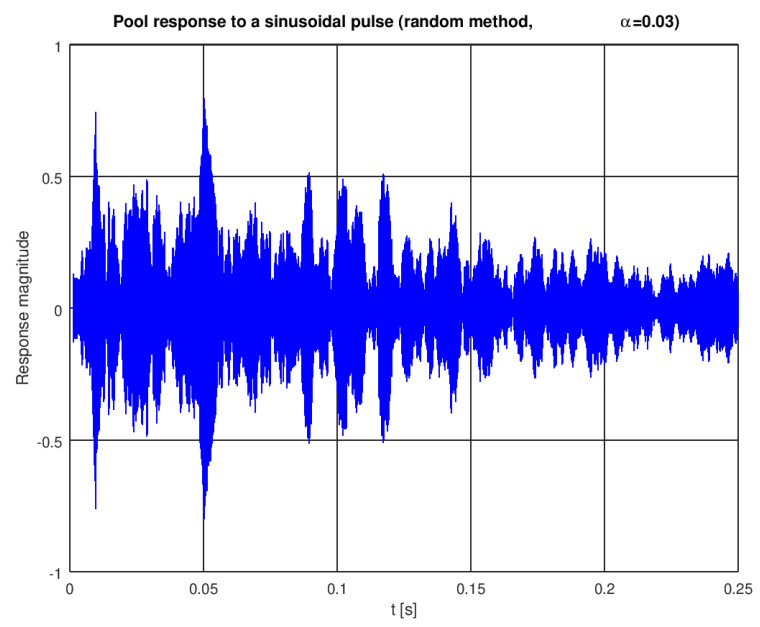
A response to a sinusoidal pulse with the model from Figure 4.

A single instance of a pool impulse response generated with the random model is shown in Figure 4. The first impulse, modeling the direct path, appears after 1393 samples. As we assumed 1 MHz sampling, this corresponds to, approximately, a 1.393 ms delay (see Figure 5 for a zoomed plot) or a 2 m path length in water. When this model is excited with a simple sinusoidal pulse (5 milliseconds of a 10 kHz sine wave), a signal shown in Figure 6 is received.

## 4. CFAR Detection in Multipath Environment

Due to external interferences from the pool equipment and users, the false alarm stabilization circuit has to be used in the detection path, very similar to the so-called CFAR technique known from radar signal processing [10]. In such a solution, the level of noise is estimated from the signal itself. Then the detection threshold is calculated as a multiple of the noise level, with a coefficient chosen to achieve the preset false alarm rate $P_{fa}$. As it is desirable to avoid false alarms, the coefficient for a pool safety system should be pretty high, typically around 17 dB. This, in turn, restricts the side lobe level of the pulse compression processor (or matched/mismatched filter). However, in a clean environment, the waveform and matched filter can be designed for a much lower side lobe level, this is not simple with multipath propagation.

**Figure 7:**
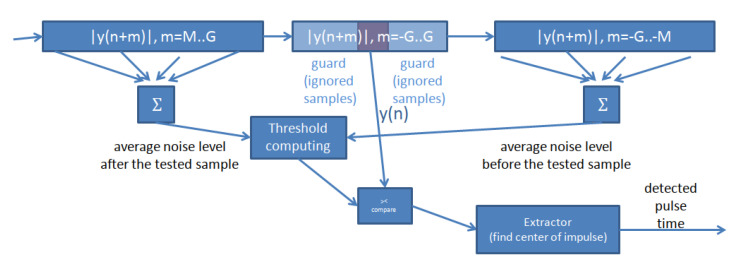
CFAR operation principle.

A typical CFAR circuit in radar (Figure 7) averages the signal level in two windows—before and after the sample under test. The sample under test and some samples around it are ignored so that the average corresponds better to noise and is not influenced by the echo peak (a “guard window”). Then, the two averages are combined to calculate the detection threshold applied to the sample under test. In radar, usually equal weights for the levels before and after the sample under test are used. Popular solutions include averaging the two values or taking the greatest of them. This approach is dictated by the assumption of a uniform spatial distribution of noise.

With a swimming pool environment, the threshold should be adapted to the noise level and not disturbed by the presence of the long “tail” caused by the echo. Thus, a CFAR circuit, in this case, should calculate the threshold based primarily on the average magnitude of samples before the sample under test. The average taken after the tested sample should be given much less weight, if not ignored.

## 5. Problem Mitigation

Besides modifying the CFAR circuit, several other techniques may be used to mitigate problems caused by multipath propagation.

### 5.1. Waveform Design

**Figure 8:**
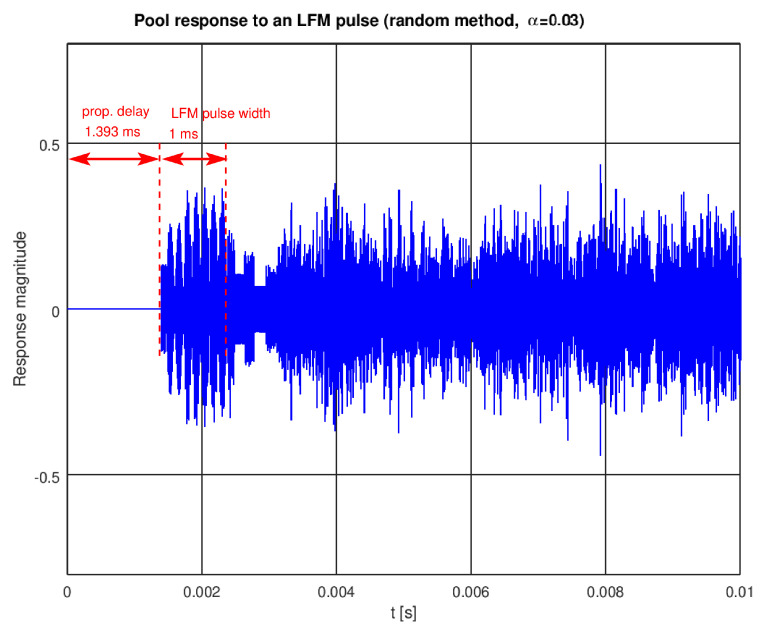
A response to an LFM pulse (chirp) with the model from Figure 4.

The energy constraints in the transmitter enforce the use of relatively long impulses for efficient signaling. Then, in a multipath environment, several copies of the signal overlay at the receiver. A known problem in such a case is selective fading—when two path lengths differ by half of the wavelength, a destructive interference happens. Thus, some frequencies in the signal may become attenuated. In the case of an unknown transmitter placement, one cannot easily predict which frequencies will suffer from the fading effect. A waveform occupying as wide a spectrum as possible is desirable, enabling successful transmission even if some frequencies are blocked. A chirp (linear frequency modulated pulse) can be a solution here, additionally benefiting from the use of a matched filter on receive. An example of model response to a chirp with the duration of 1 ms and frequency span of 50–80 kHz is presented in Figure 8.

**Figure 9:**
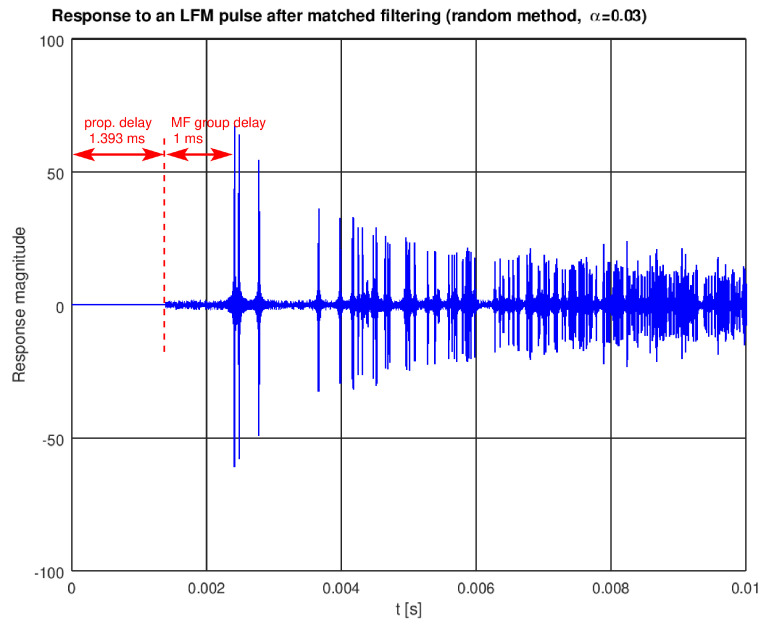
An LFM pulse response after matched filtering in the receiver.

Matched filtering (also referred to as correlation processing) used with an LFM signal not only improves the signal-to-noise ratio, but also allows separation of the paths by concentrating energy of each signal copy into a short correlation peak, as seen in Figure 9 (please note the additional 1 ms delay introduced by the filter).

### 5.2. Receiver Placement

From the schematic in Figure 3, one can easily see that a receiver located at the pool wall will suffer only from half of the reflections—the reflections from that wall (“back” paths) will either be prevented or they will yield paths of lengths equal to “front” paths. Then, locating a receiver in the pool corner might cut the number of paths again. However, such a solution should be investigated carefully, as there is a danger of creating other reflections—for example, because of wall irregularities at the corner or because technical reasons would prevent the exact corner location.

Moreover, one of the reasons for avoiding corner location is the effect of poor propagation of a signal along a pool wall—we noticed it in practical experiments several times. Thus, it may be advisable to locate sensors on different walls so that a source located close to one wall is visible from several sensors located at other walls.

### 5.3. Signal Processing

In certain circumstances, bounce paths may result in stronger echoes than the direct path signal, therefore masking the direct path correlation peak with correlation side lobes of the bounce signals. Our experiments in swimming pools show that this phenomenon frequently occurs, especially if the transmitter is not omnidirectional and is located close to a pool wall. Cancellation of bounce signals was proposed in [5]. The algorithm presented there was based on averaging of signals received with a rotating array of receivers. When the differential delay appearing due to rotation is compensated for the direct signal, the averaging causes attenuation of bounce signals reflected from the surface and the bottom of the water body. This algorithm was developed for application in a shallow oceanic water setup, and the use of such complex hardware is not reasonable in a swimming pool. Moreover, side wall bounces were not considered.

Identifying all of the critical delayed paths may allow finding the direct path peak among all correlation peaks. An algorithm for joint estimation of many path delays was presented in [4], involving a gradient-based numerical minimization in the spectral coefficient domain. Another algorithm [11] is based on the CLEAN technique, which was developed for radio astronomy, but it is commonly used in the radar community (mainly in passive radars) for finding weak echoes masked by stronger echoes. This algorithm relies on iterative removal of the echoes, starting from the most prominent one, and is numerically simpler than the joint estimation in the former algorithm. An interesting approach, employing the multipath propagation phenomenon for the improvement of the object location in a reverberant space, is described in [12]. A location accuracy down to 1/76 of the wavelength is reported. We must, however, note that exceptionally good results of this method came at the price of complicated neural network training and providing a very stable environment. This makes it hard to employ in our case, with many nuisance factors, such as people in the pool, water streams, water property variations (for example, resulting from temperature changes or air bubbles). Another variant of this approach, presented in [13], was tested in a dynamic environment. Accuracy in the wavelength range at the cost of SNR sensitivity was reported. Still, the presented approach requires a long learning process and the dynamics of the environment was constrained.

In the following, we propose a simple solution suitable for a swimming pool environment, namely a TDOA-based location algorithm that considers the hypothesis that the delays identified are not necessarily the direct-path ones.

## 6. A Multi-Hypothesis TDOA Algorithm

This section introduces a multi-hypothesis solution of the time difference of arrival (TDOA) localization problem. A standard TDOA problem setup assumes that signals arrive via the direct path from transmitter to receiver, an assumption that may be false in a swimming pool environment. Thus, we propose to amend the standard algorithm with a search for the best solution among a set of multipath reflection hypotheses.

### 6.1. Introduction to TDOA Localization

Navigation systems that use time distance of arrival (TDOA) techniques for geolocation are widely used, and this technique is widely described in the literature [14]. In the following, we summarize the TDOA problem from the perspective of source localization with multiple receivers; this problem is described with the same geometry, only the direction of signal transmission is changed.

The TDOA technique for source localization is based on measuring signal arrival times at separate receivers. As the transmit instant is assumed unknown (transmitter is not synchronized with the receivers), the time-of-flight cannot be determined—only the difference between arrival times can be used for localization (the receivers *are* synchronized with each other). When the times of arrival are converted into path lengths (actually—path length differences, calculated with respect to an arbitrary reference, e.g., the location of a selected transmitter), they are called pseudoranges.

Considering one pair of receivers, a given time difference locates a source at a hyperboloid (or a hyperbola—in a 2D case) with foci at the receivers. Thus, finding a location on a fixed plane by intersecting the hyperbolas requires a minimum of three receivers, and locating the source in 3D by intersecting hyperboloids—four receivers. For this short introduction, we skip the problem of multiple intersections and ambiguities.

**Figure 10:**
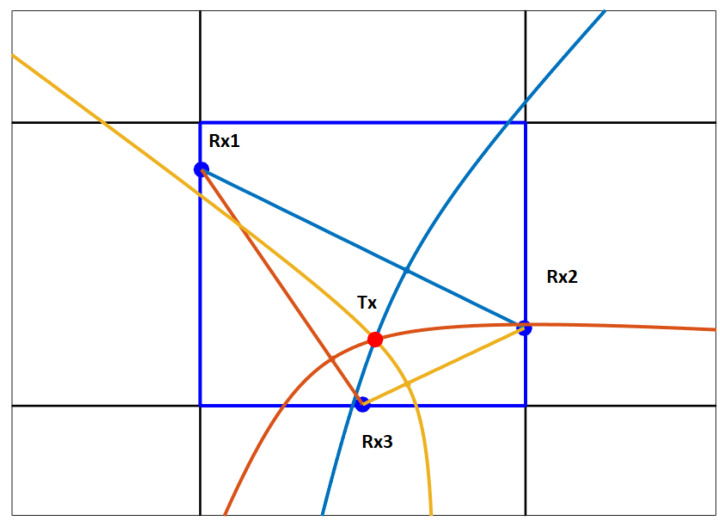
Geometric interpretation of TDOA.

An example of geometric TDOA interpretation is presented in Figure 10 (for all the drawings, we limit ourselves to a 2D problem since it is easier to present on paper). Sensors (receivers—Rx) are represented by blue dots. A red dot is the transmitter (Tx) whose position is to be calculated. The pairs of time differences are represented by distinct colors of the straight line between two sensors and a hyperbola with its focus at one of the sensors. The transmitter is therefore found at the intersection of these hyperbolas.

However, this geometric approach leads to solving a set of hyperbolic equations, which would be very hard to implement numerically. That is why other approaches have been sought. The iterative (Newton-like) error function minimization and algebraic (Bancroft) methods are among the widely known methods.

The iterative solution is based on minimizing the error in path length—i.e., comparing the differences in paths to the transmitters (pseudoranges) found from the measurement and from the source position estimate. This algorithm is most efficient when an initial position estimate is known, such as tracking a moving source. Otherwise, the number of iterations may be high, and the solution may converge to a local minimum, especially when we add constraints (e.g., saying that the source must be inside the pool area). Many versions of this algorithm exist, often combining several minimization methods—one example can be found in [15].

The algebraic method was introduced in [16], with an idea to employ the concept of a Minkowski four-dimensional space algebra. Then, after a series of calculations involving the scalar product in the Minkowski space, the problem is reduced to solving a quadratic equation. With exact time-difference measurements, one of the two roots of the quadratic indicates a true solution, and the other root gives a position that does not agree with TDOA measurements. With approximate input data, the quality of a given solution may be defined as the distance between the set of pseudoranges measured ($x_{i}$) and calculated from the found location of the source ($\hat{x_{i}}$). The distance should be calculated after removing the mean difference between sets $\bar{\hat{x_{i}}-x_{i}}$, as the solution is based on path differences. A simple formula (ignoring possible dependency between pseudorange errors) is based on the RMS difference

(6) $$q=\sqrt{1/M\sum_{m=1}^{M}{\left(\hat{x_{i}}-x_{i}-\bar{\hat{x_{i}}-x_{i}}\right)}^{2}}$$

where *M* is the number of sensors. As the value of this parameter decreases, the probability of correctness of the solution increases. This dependency allows defining a certain threshold, allowing the differentiation between a correct (or at least feasible) solution and false solution. The same parameter may be used for testing multiple hypotheses as shown in the next section.

### 6.2. Multipath Algorithm

During the tests, a situation was frequently encountered where the signal was detected in all of the receivers, but the quality of the TDOA solution was inferior, or the solution found was outside of the pool limits. After analyzing collected data, the conclusion has been reached that such a false solution is caused by an erroneous assumption of the direct signal path to the receivers. Actually, one or more paths were longer than the direct one, as the signals detected arrived with reflection from the pool wall. As described in previous chapters, the reflected signal does not fade quickly after the reflection in a pool environment. The reflected signal may happen to be much stronger than the direct one—for example, due to the directional radiation pattern of a transmitter or to some physical obstacles on a direct path.

As a result, the direct signal is masked by a stronger (reflected) signal because of the design of the CFAR processor, which, in turn, is necessary in order to keep the false alarm rate low. A wrong localization solution is an output when a path with an erroneous length is fed to the TDOA algorithm.

A multipath algorithm was designed to handle this scenario and calculate the position correctly, whose action is based on verifying multiple hypotheses regarding the presence of reflections in the propagation paths.

In the first step, we verify a simple hypothesis that assumes no reflection. We denote this as “H0”, where the number after “H” denotes the maximal number of reflections for each sensor. The multipath algorithm is used only if the H0 solution quality does not meet the expected fit quality; then the algorithm moves on to the H1 hypothesis (maximum one reflection for each sensor) and tests all of its variants. When H1 fails, more complex reflection hypotheses, H2, H3, and so on, may be tested. This approach allows avoiding excess calculation in a simple case. Each variant of the multipath hypothesis is verified based on the solution quality. The correct guess is expected to have a better fit quality than a preset threshold and to be located within the pool cuboid. The algorithm tests new hypotheses until this condition is met.

**Figure 11:**
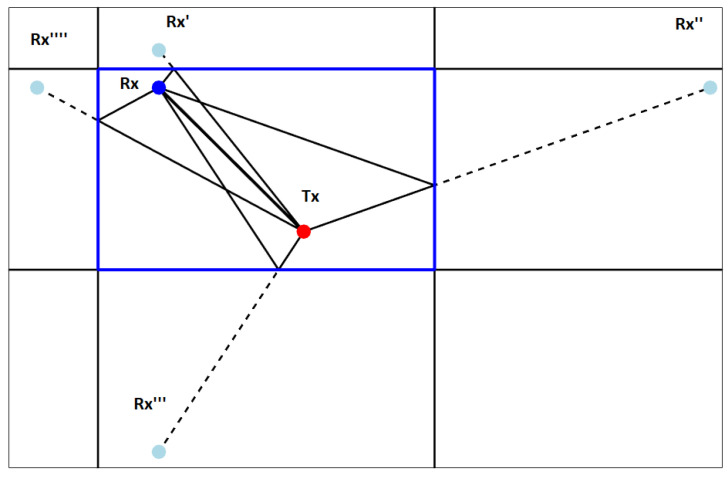
Example of possible “virtual” positions of a sensor due to reflections (2D).

To test a hypothesis with reflection, the creation of an appropriate model of the receiver geometry is required. The model can be considered dual to the propagation model presented in Figure 3, where the *source* location was reflected by pool walls—the localization model requires us to reflect the *receiver* position by the wall we assume is reflecting the wave. Once we have our model with the new “virtual” (reflected) sensors position, we can recalculate localization and verify if it is correct. The verification process considers the fit quality of the solution and the pool area limits. The example of possible reflected “virtual” positions for one sensor is presented in Figure 11.

**Figure 12:**
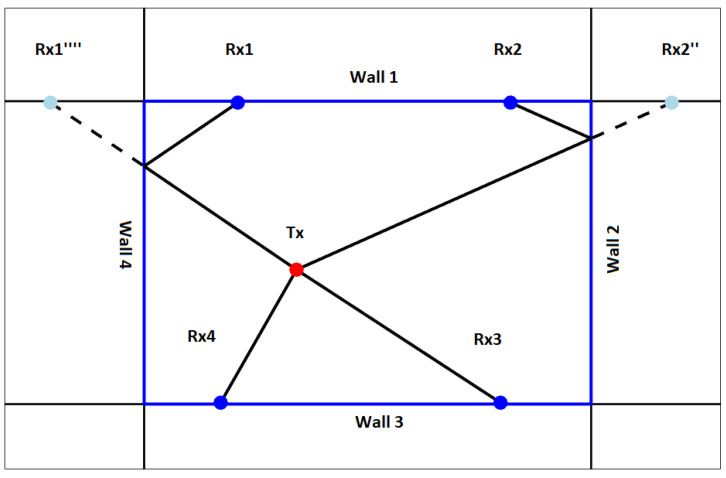
Example of handling the variant H1-4200 of reflection hypothesis H1.

In order to form all sets of given hypothesis variants, we will denote each variant of the H1 hypothesis with a string of numbers with the “H1” prefix (where “1” stands for a maximum of one reflection on each signal path). For the example presented in Figure 12, where we have four sensors and four walls, this string will contain four numbers from 0 to 4. A 0 at a given position means no reflection for the corresponding sensor, and 1–4 means that reflection occurs from the indicated wall. The numbering of walls starts with 1 and goes clockwise. The first wall has a beginning in the top left corner. The same way of numbering is assumed for sensors.

For example, the variant of H1 presented in Figure 12 will be denoted as “H1-4200”—the first sensor receives the reflection from wall number 4, second sensor—from wall number 2, third and fourth receive the direct path signal. After setting our limits and defining all possible hypotheses meeting the prerequisites, the multipath algorithm starts to iterate over all possible setups until it finds the correct one.

### 6.3. Computational Complexity

In the following, we present the computational complexity of the multi-hypothesis algorithm, expressing it as the number of basic TDOA solution calculations. Thus, testing the H0 hypothesis is presented as having the complexity equal to 1, and the complexity of testing the H1 hypothesis is equal to the number of its variants.

As we do not want to limit the algorithm to a strictly rectangular (or cuboidal, in the 3D case) pool shape, it is nearly impossible to distinguish possible and impossible reflections automatically. That is why we will count all of the variants of reflection combinations, excluding only the obviously impossible case of bouncing against the same wall twice in a row. For just one reflection (H1), the number of simple TDOA executions (localization equation solutions) to be computed can be expressed as ${\left(1+S\right)}^{N}$; for each of *N* sensors, we count the direct path and *S* possible walls with a reflection (thus, the formula also includes the H0). For the H2 hypothesis, we need to multiply the number of first reflections (*S*) by (S−1), as we exclude twice-in-a-row bounces. Then, this formula expands quickly with the subsequent hypotheses, as shown (for H>2) in Equation (7), where *H* is the hypothesis index. The actual numbers may be slightly (but not significantly) reduced if all the sensors are located at the walls—then we also exclude the last bounce against the wall where the sensor is mounted.

The results are shown in Table 1 for a four-wall (a pool simplified to a 2D shape) and six-wall (including the bottom and surface reflections) case. One can see that the complexity increases very quickly, and the time limit may be critical here—in a practical case, the algorithm has to finish its calculations before a new pulse arrives at the sensors.

(7) $$Executions\left(H,N,S\right)={\left(1+S+S\left(S-1\right)+S{\left(S-1\right)}^{2}+\ldots +S{\left(S-1\right)}^{H-1}\right)}^{N}$$

Due to the quick growth of possibilities, we decided to limit the tested hypotheses to H0 and H1 in the practical case.

### 6.4. A Real 3D Problem—Variable-Depth Bottom Reflections

A typical swimming pool is not exactly a cuboid—the pool bottom usually includes level and sloped segments to form deep and shallow water regions in the pool. Then, the bottom reflections do not follow the simple geometry of a cuboid. In the result, the inclusions of bottom reflection hypotheses consist of calculating reflections from the plane of each segment, and verifying if, for a solved location, the segment is actually able to reflect the signal in the given direction (i.e., finding the reflection point at the segment plane and testing if it is within the segment bounds). In a relatively shallow pool, an assumption of a cuboid shape may, however, lead to results that are good enough for the approximate location purpose.

A similar problem of selecting possible and impossible reflections will appear with non-rectangular pool shapes.

### 6.5. Practical Application

In the system where the algorithm found its practical application, the distress signal is transmitted in one pulse per second, and the TDOA solution must be completed before a new pulse arrives. This constraint was expected as a challenge with a resource-limited embedded computer of the system base unit. On the other hand, it is worth noticing that the reflected positions of receivers can be calculated during an initialization phase of the algorithm. Therefore, only the TDOA calculations must be performed in the run time.

The algorithm was prototyped using Octave on a PC, then implemented using C on an embedded system based on VisionSOM-6ULL module with NXP ARM Cortex-A7 processor [17]. Tests have shown that the algorithm is fully capable of working in real-time, and there is a significant time reserve before the next solution needs to be calculated.

Performance tests have shown that the C implementation of the basic TDOA solver runs approximately 40 times faster than the Octave version, and the embedded processor can calculate slightly more than 130,000 solutions per second. This is more than enough for verifying H0 and H1 but not enough for H2.

**Figure 13:**
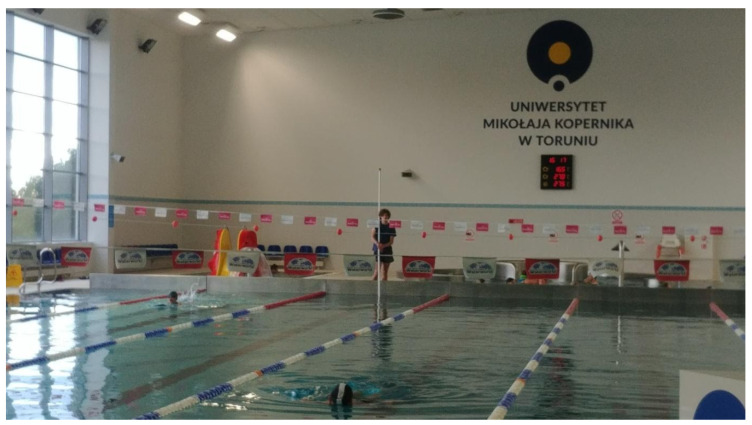
One of the authors (holding a stick with a submerged transmitter) during the system tests.

Tests were performed in several types of rectangular pools with a four-sensor system. An example photo from such tests is shown in Figure 13, where the localization solution is tested in the swimming pool of the Nicolaus Copernicus University in Toruń. The transmitter was placed on a stick under the water surface at approximately 30 cm depth, and the system calculated the source location.

**Figure 14:**
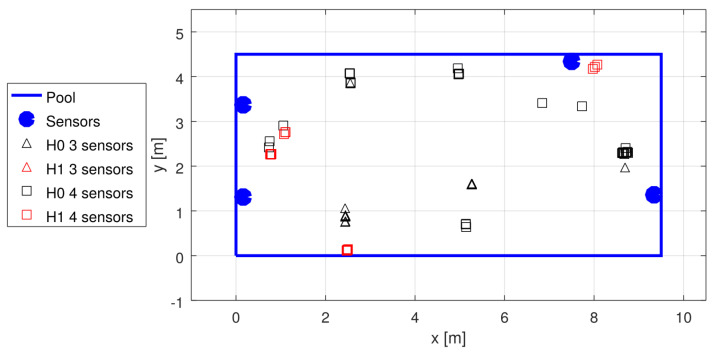
Localization results in garden pool tests.

**Figure 15:**
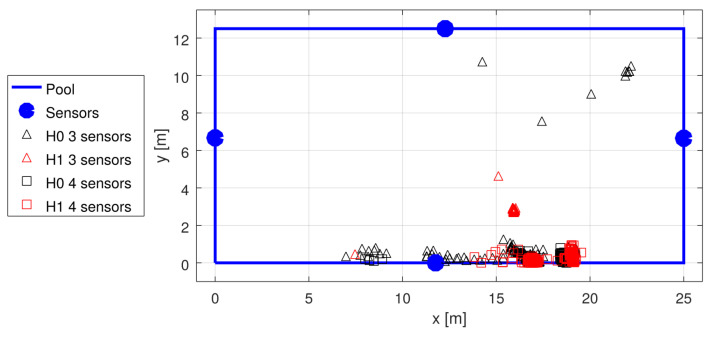
Localization results in sport pool test 1; the transmitter was moved along the pool side. A number of solutions with three sensors were accepted, but they were obviously wrong.

**Figure 16:**
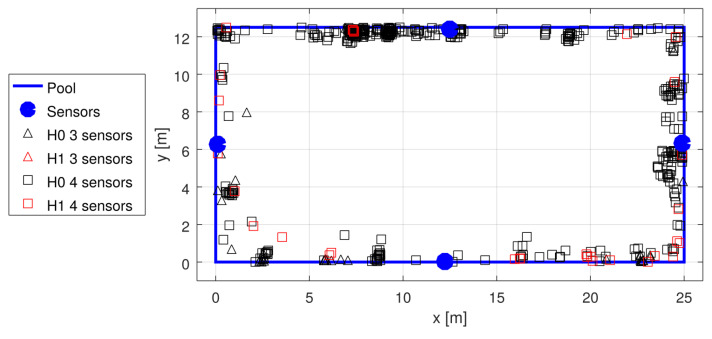
Localization results in sport pool test 2; the transmitter was moved along four sides of the pool.

**Figure 17:**
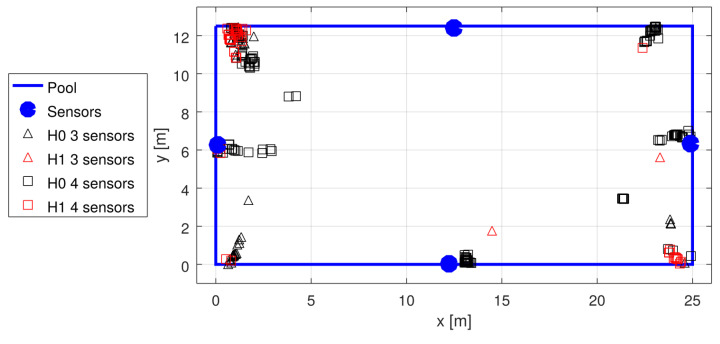
Localization results in sport pool test 3; the transmitter was placed in seven locations (close to the corners and to three sensors).

Unfortunately, the pandemic made experimenting at public pools difficult; thus, the data for the tests were scarce, and we had to rely on the available recordings from the early stages of system development. The recordings were used to generate statistics shown in Table 2. The garden pool was used first, with four sensors placed non-symmetrically (see Figure 14) at the depth of 50 cm, and with no swimmers in the pool. The three tests with a sport-type pool were conducted with 10–15 swimmers in the pool at different test campaigns, using four sensors placed in the middle of each wall, at the depth of approximately 60 cm (see Figure 15, Figure 16 and Figure 17).

The table lists the total number of detections, the numbers of accepted cases with hypotheses H0 and H1, and the number of cases when no hypothesis gave an acceptable result. The result plots show the H0 and H1 results, additionally indicating whether the detections came from three or four sensors.

In all of the tests, the algorithm was set to accept the simple hypothesis (H0) if the fit error (Equation (6)) was lower than 5 m, and the H1 was accepted only if it reduced the fit error to less than 0.5 m. The values were chosen arbitrarily; the H1 threshold was based upon the observation that a typical H1 result fulfilled this condition unless it was visibly wrong. An additional condition for accepting H0 or H1 was the location of the result within the pool.

With the tests shown, there was no “ground truth” measurement; however, one may visually spot failures, both with accepted H0 and H1 cases. An example shown in Figure 15 presents many such failures with three-sensor detections and no failures with four-sensor detections. An obvious conclusion may be that four sensors provide redundant measurements, allowing for easy verification of the solution quality (and possible rejection of the whole detection if no good solution can be found). There may, however, be a less obvious situation that one sensor may fail to detect the signal when some external interference happens, which may also impair the time measurement at other sensors.

The roughly collected statistics show that, depending on the experiment setup, in 5% to 50% of test cases, the H1 hypothesis was tried due to the failure at H0. The majority of the H1 solutions allowed correct localization of the source—especially with four-sensor detection providing some redundancy necessary for verifying the solution.

Extensive tests of the localization algorithm are planned when the circumstances allow; one of the goals will be tuning the acceptance thresholds, another goal should be determining localization accuracy with good ground truth reference measurements.

## 7. Conclusions

In this paper, we presented a geometric model of a swimming pool multipath environment. Then, we derived a randomized version of the model, which considered the non-ideality of real geometry. Finally, a multi-hypothesis algorithm was presented for locating the signal source in such an environment. The proposed algorithm is based on the geometrical model of the given pool; further research may explore an algorithm that does not depend on the cuboid-like pool shape. This may include an effort to apply machine learning techniques based on neural network training with live or simulated signals or measurements, as inspired by [12,13].

## Figures and Tables

**Table 1:** Number of executions of the basic TDOA algorithm with four and six sensors.

H	0	1	2	3	4
TDOA executions with 4 walls and 4 sensors	1	625	83,521	7,890,481	671,898,241
TDOA executions with 6 walls and 4 sensors	1	15,625	1,874,161	1,222,830,961	……
TDOA executions with 4 walls and 6 sensors	1	15,625	24,137,569	22,164,361,129	……
TDOA executions with 6 walls and 6 sensors	1	117,649	2,565,726,409	……	……

**Table 2:** Practical test results.

Pool Type	Pool Size	Total Cases	H0 Cases	H1 Cases	Rejected Cases
Garden pool	9.5 m × 4.5 m	187	91	96	0
Sport pool test 1	25 m × 12.5 m	716	442	262	12
		Note: from visual verification of the results (Figure 15) 8 out of H0 cases and 20 out of H1 cases are wrong			
Sport pool test 2	25 m × 12.5 m	711	649	33	29
Sport pool test 3	25 m × 12.5 m	345	266	61	18
